# Unrecognized, Undiagnosed, and Untreated: Cardiac-Disease-Induced PTSD among Patients' Partners

**DOI:** 10.3389/fpsyg.2017.01265

**Published:** 2017-07-25

**Authors:** Noa Vilchinsky

**Affiliations:** Department of Psychology, Bar-Ilan University Ramat Gan, Israel

**Keywords:** acute coronary event, cardiac-disease-induced-PTSD, caregivers, intimate partners, cardiovascular diseases

The experience of cardiac patients' family members and especially patients' partners—who are potential trauma victims—is an issue which has received almost no attention in the literature. I wish to draw attention to this fairly substantial at-risk population, a population which has been neglected both in the trauma research as well as by practitioners.

Cardiovascular disease (CVD) is a leading cause of death worldwide (Centers for Disease Control and Prevention, 2016). Each year CVD causes over four million deaths in Europe (European Heart Network and European Society of Cardiology, 2012), and during that same time period about 735,000 Americans undergo a heart attack (Centers for Disease Control and Prevention, 2016). Despite growing survival rates (National Institutes of Health, 2010; Central Bureau of Statistics, 2012), cardiac diseases are still regarded as a significant threat to one's life and can lead to, among other things, severe mental states as depression and anxiety (Fisher and Collins, 2012).

Cardiac events, and especially acute ones—are often marked by features that are particularly traumatizing. Among these are the event's abruptness, the real and present danger of death indicated by the event, and the intense sense of loss of control and helplessness that patients experience during the event (Kutz et al., 1994; Edmondson, 2014). Indeed, in the past 20 years, much of the data that has emerged indicates that the post-traumatic stress symptoms (PTSS) experienced by some cardiac patients actually constitute a diagnosis of post-traumatic stress disorder (PTSD). Studies suggest that 12–15% of all patients who undergo an acute coronary event (ACE) subsequently develop cardiac-disease-induced PTSD (CDI-PTSD) (Edmondson, 2014).

According to the DSM-5, individuals are susceptible to PTSD not only when they undergo an event but also when they witness “in person, the event(s) as it occurred to others” or when they learn “that the traumatic event occurred to a close family member or close friend…” (American Psychiatric Association, 2013, p. 271). As the average age of cardiac disease onset is late adulthood, it is the patient's partner who is most vulnerable to CDI-PTSD, since the primary caregiver at this time of life is usually the spouse. A patient's partner might very well have witnessed the cardiac event, a factor which differentiates this kind of traumatic event from others in which the partner would not generally be present—i.e., war, captivity, or sexual assault. According to the 2010 ACSIS (Acute Coronary Syndrome Israeli Survey) survey conducted in Israel (ACSIS-Israel Center for Disease Control, 2011), 77.5% of all ACE patients have experienced the onset of their cardiac event at home, making it quite likely that their partners were also present for it. In addition, 49.1% of all ACE patients arrived independently and/or in their private cars at the hospital, again suggesting that their partners, who probably aided in transporting them to the hospital, were very much involved in at least some aspects of the event.

Regardless of whether they actually witnessed the cardiac event, cardiac partners are usually the ones most “on the scene” with their loved ones, both literally as they usher them through their stay in the Cardiac Care Unit, as well as more figuratively when providing them with tangible and emotional support (e.g., Delon, 1996; Randall et al., 2009). It is therefore reasonable to suggest that partners of cardiac patients are highly susceptible to developing CDI-PTSD themselves.

Despite the above, almost no data are available on the existence of CDI-PTSD among patients' partners. In fact, until 2016 there have only been four studies assessing the prevalence of CDI-PTSD among partners of cardiac patients, and these studies focused only on heart transplant patients. Specifically, Bunzel et al. (e.g. Bunzel et al., 2005) found that 23–25% (*n* = 6) of patients' partners (all women) met the criteria for CDI-PTSD, whereas none of the patients did. Partners showed a higher CDI-PTSD prevalence at the 6-month follow-up (14%), according to Brouwers et al. (2013), than did patients (9%). Finally, Stukas et al. (1999) identified only two dyads in which both partner and patient were classified as having definite or probable PTSD.

Despite the fact that only 5,000 heart transplantations are conducted annually worldwide, compared with 735,000 new ACE cases every year in the USA alone, only one study to date has assessed the prevalence of CDI-PTSD among partners of ACE patients. In this study, conducted in Israel, we detected that whereas 13% of our cardiac patients were screened for PTSD, a full 25% of their partners met the same screening criteria (Fait et al., 2016).

Thus, more attention should be directed into the *partners* of patients diagnosed with ACE. For example, partners' post-traumatic symptoms and well-being should be evaluated as an integral part of cardiac prevention and rehabilitation programs. Tailored intervention programs to alleviate partners' post-traumatic stress should be developed, and their effectiveness should be assessed, in order to help partners overcome this major medical crisis in their families.

More research is needed in order to establish crucial issues such as the exact timing of the emergence of partners' CDI-PTSD; risk factors, as well as buffers, for partners' CDI-PTSD; and psychological, physiological, and behavioral consequences of partners' CDI-PTSD for both partners and patients. Since partners of cardiac patients most often act as their primary caregivers, it is important to identify how partners' CDI-PTSD may hinder their ability to provide effective support for the patients. In addition, studies need to focus on the consequences of CDI-PTSD for the couple relationship as well as for other family members such as children. Finally, partners' CDI-PTSD is still to be established as a valid clinical diagnostic entity.

In sum, being exposed to a patient's acute coronary event, in combination with serving as the patient's primary caregiver, may very well make patients' partners candidates for developing CDI-PTSD themselves. Unfortunately, the research into CDI-PTSD among cardiac patients' partners is severely lacking. From a clinical point of view, given the absolute number of cardiac-disease patients worldwide, a substantial number of individuals with posttraumatic stress symptoms may have gone unrecognized, undiagnosed, and untreated.

## Author contributions

The author confirms being the sole contributor of this work and approved it for publication.

### Conflict of interest statement

The author declares that the research was conducted in the absence of any commercial or financial relationships that could be construed as a potential conflict of interest.
